# Management of Retained Intervention Guide-wire: A Literature Review

**DOI:** 10.2174/1573403X11309030010

**Published:** 2013-08

**Authors:** Abdulrahman M Al-Moghairi, Hussein S Al-Amri

**Affiliations:** Department of Adult Cardiology, Prince Sultan Cardiac Center (PSCC), Riyadh, Saudi Arabia

**Keywords:** Coronary Angioplasty, Guide-wire Fracture, Guide wire Entrapment, Retained guide wire remnant, Guide wire Retrieval.

## Abstract

Percutaneous coronary angioplasty is increasingly employed in the treatment of patients with complex coronary artery disease.

Different steerable guide wires used to open occluded vessel and facilitate balloon and stent deployment. However, the guide-wire itself is not without hazard: it may perforate or dissect the vessel, but fracture or entrapment is uncommon. Its management depends on the clinical situation of the patient, as well as the position and length of the remnant.

In this review we discuss the angioplasty guide-wire fracture and entrapment risk factors, potential risks and management.

## INTRODUCTION

Entrapment and fracture of coronary guide-wire is a rare complication of percutaneous coronary interventions (PCI). The incidence of these complications is approximately 0.1- 0.2 % [1, 2]. Entrapment or over-rotation of the distal tip of the angioplasty guide wire can lead to the wire rupture [3]. Excessive bending produces a high tensile load to the guide-wire, especially when applied to the junction point between the very flexible distal 3-cm tip and the remainder of the guide wire, may result in wire fracture [4]. Retention of hardware components in the coronary tree has been recently reported to complicate coronary angioplasty [1, 5]. 

The management of patients with retained catheter or wire fragments within the coronary artery tree is difficult. Small fractured components can be left within a chronically occluded coronary artery without sequelae [1, 6, 7]. Since intravascular wire fragments are highly thrombogenic, immediate surgical removal, eventually combined with bypass grafting may be indicated if percutaneous retrieval is unsuccessful or difficult [8]. 

Here, we review the literature for published data in English about entrapped angioplasty wire and summarize the management options available.

## METHODS

We searched the Medline (PubMed), Embase, EBSCO, ScienceDirect and Cochrane databases for published data or reports in English from 1980 to 2012 using the Medical Subject Heading terms “coronary guide-wire fracture, entrapment, unraveling, or retained guide-wire fragments.”

## RESULTS

A literature search revealed a total of 67 patients in 48 reports with guide-wire entrapment and different management approaches which involved percutaneous and surgical retrieval of entrapped fragments and conservative therapy for some cases Table **1**. 

The wire entrapment was reported in the right coronary artery (RCA) in 22 cases, left anterior descending artery (LAD) in 25 cases, left circumflex artery (LCX) in 19 cases and ramus intermedius artery (RI) in 2 cases.

The surgical extraction was performed in 29 cases (43.3%) and percutaneous therapy used in 28 cases (41.8%), while 10 cases (14.9%) received conservative therapy [1, 3, 9-14] Fig. (**1**). Interestingly, floppy wires were used in most of the cases.

Several percutaneous techniques used for retrieval of entrapped guide-wire fragments including stenting against the vessel wall (7 cases) [12, 15-19], snare loop (9 cases) [1, 20-25], double or triple wire technique (3 cases) [7, 26, 27], bioptome (1 case) [1], tornus micro-catheter (1 case) [28], deep-guide catheter wedging with balloon inflation (6 cases) [1, 3, 29, 30] and pigtail catheter (1 case) [6]. 

## DISCUSSION

### Prevalence of Coronary Guide Wire Fracture

Hartzler and colleagues reported angioplasty guide-wire retention in 8 cases of 5,400 consecutive Percutaneous transluminal coronary angioplasty (PTCA) procedures, 4 patients with retained wire segment treated conservatively had no clinical sequel on long-term follow-up [1]. The broken or retained guide-wire is a rare complication of angioplasty procedures, with an estimated incidence of 0.1 - 0.2 % [1, 2]. 

### Risk Factors for Guide Wire Fracture

The possible mechanics of the rupture of these delicate, soft wires entails several factors: the usual practice of PTCA is to advance the wire across the stenotic lesion for a distance to facilitate guidance of the balloon easily across the stenosis. The guide-wire is rotated during advancement to negotiate the correct course. This rotational maneuver should never exceed 180 degrees. Excessive rotation, especially if the tip is not free, leads to lateral stress caused by torqueing and unraveling of the platinum coil and precipitates rupture [31].

### Risk of Retained Guide Wire Filament

The guide-wire remnants can lead to complications, such as perforation, thrombosis, embolic phenomena and vessel occlusion [5, 8, 31]. 

### Rationale of Guide Wire Fragments Extraction

Since the guide-wire is thrombogenic and its presence inside the coronary or hanging up into the aorta may carry a risk of thrombo-embolization, this makes the fragment removal is essential to minimize this risk.

## MANAGEMENT STRATEGIES OF RETAINED GUIDE WIRE REMNANTS

### General Considerations of Retrieval

The percutaneous coronary intervention is usually completed uneventfully, with satisfactory results for the operator and the patient. Complications are unusual but when they do occur the sequences are serious. The operator should understand how to deal with them. One such complication is the guide-wire entrapment and the decision-making depends on whether the wire is still intact or fractured, and the site and extent of entrapment. The choice of guide catheter for more effective support is a crucial step and, given the prolonged nature of retrieval procedure, meticulous attention should be paid to ensure adequate anticoagulation. 

In a case of guide-wire fracture, three therapeutic options are considered: percutaneous retrieval, surgical removal, or leaving the corpus alienum in-situ. The most elegant one is the non-surgical procedure by capturing the fragment depending on the operator`s comfort and experience. However, this approach carries the risk of additional vascular trauma, coronary spasms, or new fragmentation. If the removal by catheter fails and/or local myocardial ischemia arises with or without circulatory instability, and especially when extravasation of contrast medium gives evidence of vessel laceration urgent operation is indicated. Table **2** summarizes the possible methods of extraction of the retained guide-wire fragments.

### Catheter Based Retrieval

There is no device designed for fractured wire retrieval. Retrieval can be attempted using a further two or more wires passed alongside the entrapped wire, and the torque is then applied to all wires and a twisting action results in wires wrapping around the retained wire and trapping it between the wrapped portions. The twisted group is then retracted, pulling out the entrapped wire out of coronary towards the guide catheter then externalization of the catheter and the wires as one unit [7, 26, 27]. 

A deep-guide catheter wedge and balloon inflation technique is another method by which the entrapped wire can be retrieved. This is a method used if the wire is still intact and the guide is over-wedged, then the balloon is advanced and inflated at the terminal part of the guide catheter and is tightly trapping the wire and the whole system is retracted to pull out the retained wire [1, 3, 29, 30]. Another method to free the retained wire is the use of tornus micro-catheter, in which the micro-catheter is advanced with particular rotations to the tip of the wire to allow for the release of the jailed or entrapped part [28]. 

The use of a snare loop to retrieve the entrapped guide-wire fragment was successful in some cases, but the snare may not match the vessel diameter. The gooseneck snare was the most commonly used technique and more suitable for proximal, large size vessels.

If the wire tip could not be freed and the retained fragment is entirely inside the branch, then stenting against the vessel wall might be the option [12, 18]. Percutaneous methods of retrieval are listed in Table **3**.

### Surgical Extraction

If percutaneous techniques fail, surgery is warranted. Immediate surgical removal, eventually combined with bypass grafting, should be done. However, the unplanned cardiac surgery is associated with significant morbidity and mortality. Several surgical approaches had been reported for the treatment of retained guide-wire fragments. Bypass surgery is performed in most of the cases. Surgical extraction includes direct coronary arteriotomy or aortotomy [2-5, 8, 29, 31-47]. 

Left Main (LM) coronary arteriotomy and patch repair has been used for proximal wire entrapment [48]. 

### Medical Management

The attempt to remove retained guide-wire remnants from coronary circulation is the preferable option. Some case reports and case series suggested that in selected patients, a reasonable option might be to leave the guide-wires in-situ without attempting extraction techniques if there is a chance of success seems remote based on the anatomic and technical considerations [1]. Treatment of such patients with systemic anticoagulation and anti-platelets agent with close follow up appears more appropriate for occluded or smaller distal vessels and early surgical referral if ischemic events are encountered. 

### Complications of Guide Wire Retrieval

Prolonged manipulation of retrieval devices or catheters may increase the risk of thrombus or air embolization. Failure of removal of retained fragments may lead to myocardial ischemia due to coronary thrombosis or obstruction. Vessel dissections or rupture from repeated instrumentation may lead to tamponade or emergency cardiac surgery with associated high mortality. 

## CONCLUSION

Intervention guide-wire fracture and entrapment is a rare complication of coronary interventions. The operators should be aware of this complication and be familiar with the measures to avoid it and to appropriately manage it.

## Figures and Tables

**Fig. (1):**
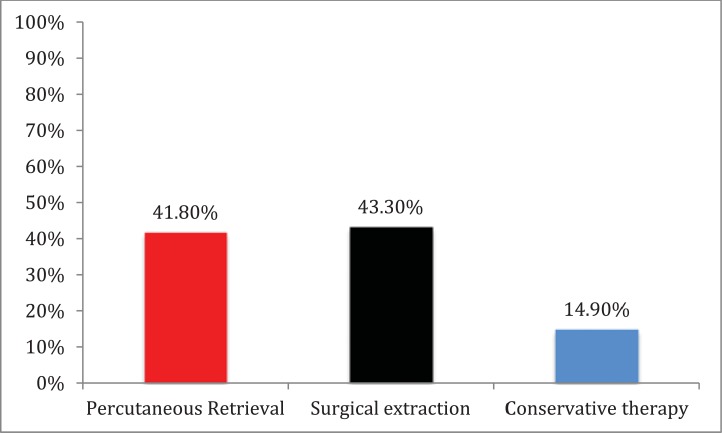
Management approaches used for entrapped wire fragment.

**Table 1.** List of the Published Reports in English About Guide Wire Entrapment

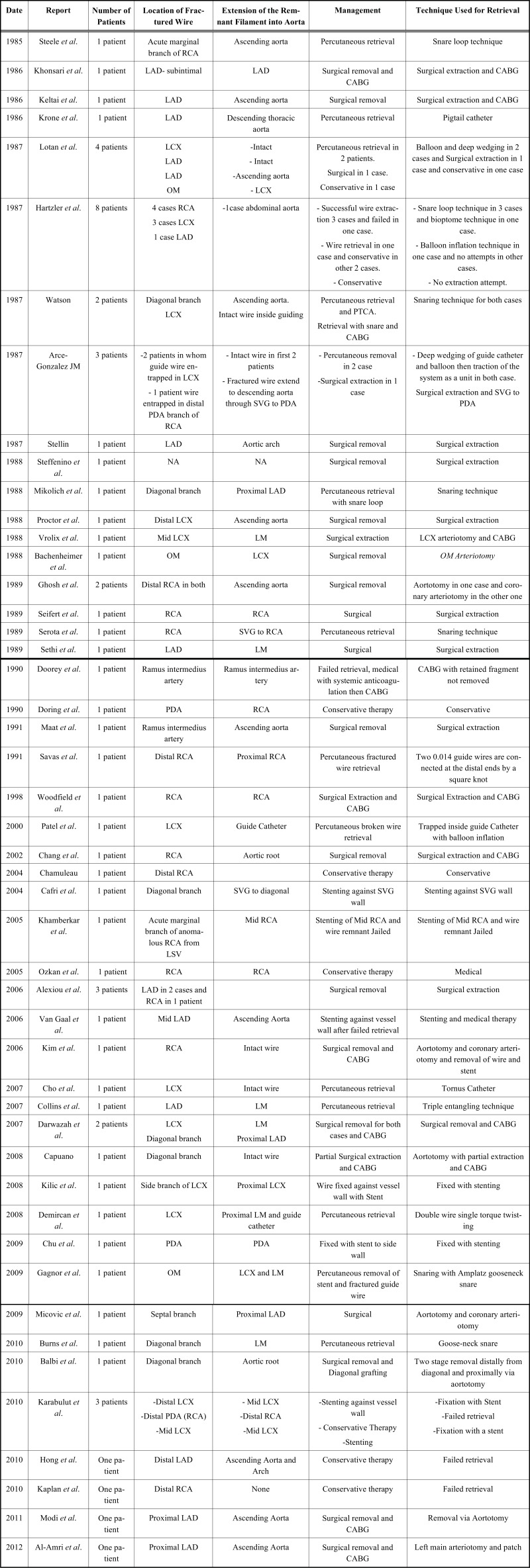

**Table 2.** Methods of Extraction of Retained Guide Wire Fragment

**A. Percutaneous Methods** Double or triple wire technique Deep wedging of guiding catheter and traction of the system Retrieval using Balloon inflation technique Retrieval by snare loop Retrieval using micro catheter e.g. Tornus catheter Extraction with Bioptome Stenting against vessel wall
**B. Surgical extraction**
**C. Conservative therapy**

**Table 3.** The Percutaneous Techniques used to Retrieve Entrapped Wire Fragment

Modality	Number of case (%) Total = 28
Snare loop	9 (32.1)
Double or triple wire technique	3 (10.7)
Deep guide catheter wedge with balloon inflation	6 (21.4)
Tornus micro-catheter	1 (3.6)
Pigtail catheter	1 (3.6)
Stenting against vessel wall	7 (25)
Bioptome	1 (3.6)

## ABBREVIATIONS

LM: = Left Main coronary artery
LAD: = Left Anterior Descending artery
LCX: = Left Circumflex artery
OM: = Obtuse Marginal branch
PDA: = Posterior Descending Artery
RI: = Ramus Intermedius artery
RCA: = Right Coronary Artery
SVG: = Saphenous vein graft
PCI: = Percutaneous Coronary Interventions
PTCA: = Percutaneous transluminal coronary angioplasty

## References

[1] Hartzler GO, Rutherford BD, McConahay DR (1987). Retained percutaneous transluminal coronary angioplasty equipment components and their management. Am J Cardiol.

[2] Steffenino G, Meier B, Finci L (1988). Acute complications of elective coronary angioplasty: a review of 500 consecutive procedures. Br Heart J.

[3] Lotan C, Hasin Y, Stone D, Meyers S, Applebaum A, Gotsman MS (1987). Guide wire entrapment during PTCA: a potentially dangerous complication. Catheterization and cardiovascular diagnosis.

[4] Vrolix M, Vanhaecke J, Piessens J, De Geest H (1988). An unusual case of guide wire fracture during percutaneous transluminal coronary angioplasty. Catheterizat Cardiovasc Diagnosis.

[5] Khonsari S, Livermore J, Mahrer P, Magnusson P (1986). Fracture and dislodgment of floppy guidewire during percutaneous transluminal coronary angioplasty. Am J Cardiol.

[6] Krone RJ (1986). Successful percutaneous removal of retained broken coronary angioplasty guidewire. Catheterizat Cardiovasc Diagnosis.

[7] Savas V, Schreiber T, O'Neill W (1991). Percutaneous extraction of fractured guidewire from distal right coronary artery. Catheterizat Cardiovasc Diagnosis.

[8] Keltai M, Bartek I, Biro V (1986). Guidewire snap causing left main coronary occlusion during coronary angioplasty. Catheterizat Cardiovasc Diagnosis.

[9] Doring V, Hamm C (1990). Delayed surgical removal of a guide-wire fragment following coronary angioplasty. Thoracic Cardiovasc Surgeon.

[10] Chamuleau SAJ (2004). S-TJM: 'Lost and found': a coronary guidewire
remnant. Netherland Heart J.

[11] Ozkan M, Yokusoglu M, Uzun M (2005). Retained percutaneous transluminal coronary angioplasty guidewire in coronary circulation. Acta Cardiol.

[12] Karabulut A, Daglar E, Cakmak M (2010). Entrapment of hydrophilic coated coronary guidewire tips: which form of management is best?. Cardiol J.

[13] Hong YM, Lee SR (2010). A case of guide wire fracture with remnant filaments in the left anterior descending coronary artery and aorta. Korean Circulat J.

[14] Kaplan S, Kaplan ST, Kutlu M (2010). An unusual case of guide wire fractured during primary percutaneous coronary intervention, and two year follow-up. Kardiologia polska.

[15] Cafri C, Rosenstein G, Ilia R (2004). Fracture of a coronary guidewire during graft thrombectomy with the X-sizer device. J Invasive Cardiol.

[16] Khambekar S, Hudson I, Kovac J (2005). Percutaneous coronary intervention to anomalous right coronary artery and retained piece of guidewire in the coronary vasculature. J Intervent Cardiol.

[17] van Gaal WJ, Porto I, Banning AP (2006). Guide wire fracture with retained filament in the LAD and aorta. Intern J Cardiol.

[18] Kilic H, Akdemir R, Bicer A (2008). Rupture of guide wire during percutaneous transluminal coronary angioplasty, a case report. Intern J Cardiol.

[19] Chu CY, Lin TH, Su HM, Voon WC, Lai WT, Sheu SH (2009). Management of a retained coronary guidewire fragment during percutaneous transluminal coronary angioplasty: a case report. Kaohsiung J Med Sci.

[20] Steele PM, Holmes DR, Mankin HT, Schaff HV (1985). Intravascular retrieval of broken guide wire from the ascending aorta after percutaneous transluminal coronary angioplasty. Catheterizat Cardiovasc Diagnosis.

[21] Watson LE (1987). Snare loop technique for removal of broken steerable PTCA wire. Catheterizat Cardiovasc Diagnosis.

[22] Mikolich JR, Hanson MW (1988). Transcatheter retrieval of intracoronary detached angioplasty guidewire segment. Catheterizat Cardiovasc Diagnosis.

[23] Serota H, Deligonul U, Lew B, Kern MJ, Aguirre F, Vandormael M (1989). Improved method for transcatheter retrieval of intracoronary detached angioplasty guidewire segments. Catheterizat Cardiovasc Diagnosis.

[24] Gagnor A, Tomassini F, Infantino V, Varbella F (2009). Unintended stent removal during fractured-guidewire removal in emergency angioplasty. J Cardiovasc Med (Hagerstown).

[25] Burns AT, Gutman J, Whitbourn R (2010). Side-branch wire entrapment
during bifurcation PCI: avoidance and management. Catheterization and cardiovascular interventions : J Society
Cardiac Angiograph Intervent.

[26] Collins N, Horlick E, Dzavik V (2007). Triple wire technique for removal of fractured angioplasty guidewire. J Invasive Cardiol.

[27] Demircan S, Yazici M, Durna K, Yasar E (2008). Intracoronary guidewire
emboli: a unique complication and retrieval of the wire. Cardiovasc
Revascularizat Med: including molecular interventions.

[28] Cho YH, Park S, Kim JS (2007). Rescuing an entrapped guidewire using a Tornus catheter. Circulation.

[29] Arce-Gonzalez JM, Schwartz L, Ganassin L, Henderson M, Aldridge H (1987). Complications associated with the guide wire in percutaneous transluminal coronary angioplasty. J Am College Cardiol.

[30] Patel T, Shah S, Pandya R, Sanghvi K, Fonseca K (2000). Broken
guidewire fragment: a simplified retrieval technique. Catheterizat
Cardiovasc Intervent: official journal of the Society for Cardiac
Angiography & Interventions.

[31] Ghosh PK, Alber G, Schistek R, Unger F (1989). Rupture of guide wire
during percutaneous transluminal coronary angioplasty. Mechanics
and management. J Thoracic Cardiovasc Surgery.

[32] Sethi GK, Ferguson TB, Miller G, Scott SM (1989). Entrapment of broken guidewire in the left main coronary artery during percutaneous transluminal coronary angioplasty. Annals Thoracic Surgery.

[33] Stellin G, Ramondo A, Bortolotti U (1987). Guidewire fracture: an unusual complication of percutaneous transluminal coronary angioplasty. Intern J Cardiol.

[34] Proctor MS, Koch LV (1988). Surgical removal of guidewire fragment following transluminal coronary angioplasty. Annals Thoracic Surgery.

[35] Bachenheimer LC, Green CE, Rosing DR, Wallace RB (1988). Surgical removal of the intracoronary portion of a fractured angioplasty guidewire. Am J Cardiol.

[36] Seifert PE, Auer JE (1989). Removal of guidewire fragment. Annals Thoracic Surgery.

[37] Doorey AJ, Stillabower M (1990). Fractured and retained guide-wire fragment during coronary angioplasty--unforeseen late sequelae. Catheterizat Cardiovasc Diagnosis.

[38] Maat L, van Herwerden LA, van den Brand M, Bos E (1991). An unusual problem during surgical removal of a broken guidewire. Annals Thoracic Surgery.

[39] Woodfield SL, Lopez A, Heuser RR (1998). Fracture of coronary guidewire during rotational atherectomy with coronary perforation and tamponade. Catheterizat Cardiovasc Diagnosis.

[40] Chang TM, Pellegrini D, Ostrovsky A, Marrangoni AG (2002). Surgical
management of entrapped percutaneous transluminal coronary
angioplasty hardware. Texas Heart Institute journal / from the
Texas Heart Institute of St Luke's Episcopal Hospital, Texas
Children's Hospital.

[41] Alexiou K, Kappert U, Knaut M, Matschke K, Tugtekin SM (2006). Entrapped coronary catheter remnants and stents: must they be
surgically removed?. Texas Heart Institute journal / from the Texas
Heart Institute of St Luke's Episcopal Hospital, Texas Children's
Hospital.

[42] Kim CK, Beom Park C, Jin U, Ju Cho E (2006). Entrapment of guidewire in the coronary stent during percutaneous coronary intervention. Thoracic Cardiovasc Surgeon.

[43] Darwazah AK, Abu Sham'a RA, Yassin IH, Islim I (2007). Surgical intervention to remove an entrapped fractured guidewire during angioplasty. J Cardiac Surgery.

[44] Capuano F, Simon C, Roscitano A, Sinatra R (2008). Percutaneous transluminal coronary angioplasty hardware entrapment: guidewire entrapment. J Cardiovasc Med (Hagerstown).

[45] Micovic SV, Nezic D, Mangovski L, Djukanovic B, Vukovic P (2009). Coronary-coronary bypass to reconstruct coronary artery bed following removal of a guidewire entrapped in a stent. Thoracic Cardiovasc Surgeon.

[46] Balbi M, Bezante GP, Brunelli C, Rollando D (2010). Guide wire fracture
during percutaneous transluminal coronary angioplasty: possible
causes and management. Interactive Cardiovasc Thoracic Surgery.

[47] Modi A, Zorinas A, Vohra HA, Kaarne M (2011). Delayed surgical retrieval of retained guidewire following percutaneous coronary intervention. J Cardiac Surgery.

[48] Al-Amri HS, Al-Moghairi AM, Calafiore AM (2012). Left Main Approach for Retrieval of Retained Guidewire Fragment. J Cardiac Surgery.

